# Prevalence of latent tuberculosis in patients with hematological neoplasms in a cancer referral hospital in Mexico City

**DOI:** 10.1186/s12879-021-06236-y

**Published:** 2021-05-31

**Authors:** Erick Antonio Osorio-López, Diana Vilar-Compte, Jaquelyn García-Tirado, Alexandra Martin-Onraet

**Affiliations:** 1 Faculty of Medicine, National Autonomous University of Mexico (Universidad Nacional Autonoma de México), Mexico City, Mexico; 2 Department of Infectious Diseases, National Cancer Institute (Instituto Nacional de Cancerologia), Av. San Fernando 22, col. Sección XVI. 14000, Tlalpan Delegation, Mexico City, Mexico

**Keywords:** Latent tuberculosis, Hematologic malignancies, Tuberculin skin test

## Abstract

**Objective:**

To determine the prevalence of Latent Tuberculosis in patients with hematological neoplasms at the Instituto Nacional de Cancerología in Mexico City using the Tuberculin skin test (TST).

**Methods:**

This retrospective study included all patients with a recent diagnosis of hematological neoplasms who were admitted for treatment from 2017 to 2018 and who were screened for latent tuberculosis with the TST. The prevalence of latent tuberculosis in this group, tolerance and therapeutic adherence in treated patients are described.

**Results:**

The files of 446 patients with hematological malignancy who had a TST were reviewed. The prevalence of latent tuberculosis was 31.2% (*n* = 139). Ninety-three patients received isoniazid, 15.1% had some adverse reactions, but only 4 (4.3%) had to discontinue treatment. Two patients with latent tuberculosis under treatment with Isoniazid reactivated tuberculosis infection.

**Conclusions:**

The prevalence in our study was within the range of other similar Mexican populations. Isoniazid treatment had an adequate tolerance and adherence. Longer follow-up could offer more information on the risk of reactivation in both groups.

## Introduction

Tuberculosis has a major impact on the burden of disease globally. Approximately a quarter of the world’s population is infected with *Mycobacterium tuberculosis* [1, 2] and its distribution is mainly in low- and middle-income countries, such as Mexico [3]. In Mexico, in 2019, an incidence of 23 cases per 100,000 inhabitants was documented [4].

After exposure to the tuberculosis bacillus, the majority of immunocompetent people will achieve immunological containment, remaining in a silent state called latent tuberculosis (LTBI), characterized by the absence of clinical manifestations [1, 3]. However, under conditions of immunosuppression and/or in case of not receiving prophylactic treatment, it can reactivate and progress to “active” tuberculosis [1, 5–7].

The vast majority of active tuberculosis cases are due to the reactivation of a primary infection [1, 3, 8]. In the general population, a patient with LTBI has a 10% risk of reactivation of tuberculosis, and half of these reactivations occur in the first 2 years of infection [5, 9]. In immunosuppressed patients this risk increases [9, 10], mainly in those with hematological neoplasms. For this reason, it has been recommended to screen for LTBI in immunosuppressed patients and to assess treatment in those who test positive, to reduce the probability of reactivation of tuberculosis [1, 5, 11, 12].

The most widely used diagnostic tool for the detection of LTBI is the tuberculin skin test (TST) [2, 3, 13]. Another method approved by international guidelines is IFNγ release assays (IGRAs), which offer greater specificity. However, both tests have decreased sensitivity in immunosuppressed patients. Detection algorithms have been proposed using the two tests consecutively to increase sensitivity or applying a TST booster. Until now there is little information reported in this regard in patients with hematological neoplasms. A recent Mexican study reported a 20% prevalence in bone marrow transplant candidates who underwent TST booster screening [14].

Treatment for LTBI has been shown to be effective in preventing its progression to active TB in immunocompromised patients [15–18]. The currently recommended regimens are isoniazid (INH) 5 mg/kg once a day for 9 months [1], rifampin once a day for 4 months, or combined treatments (isoniazid plus rifapentine or isoniazid plus rifampicin for 3 months) [1, 9].

In Mexico, there are few publications on the prevalence of LTBI in patients with hematologic malignancies. The Instituto Nacional de Cancerología (INCan) has a large population of cancer patients. The aim of this study was to establish the prevalence of LTBI in patients with a recent diagnosis of hematological neoplasm and tolerance to treatment with isoniazid in our Institution.

## Material and methods

INCan is a cancer, referral and teaching hospital located in Mexico City, which serves adult cancer patients who do not have social security, mainly from the central region of the country. It has 133 beds and a 40-bed hematology hospitalization service.

### Screening for latent tuberculosis

As of 2017, the INCan routinely applied the detection and treatment of LTBI in patients with hematological neoplasms, as recommended by international guidelines. As part of the initial studies in patients with suspected hematologic malignancy, screening for latent tuberculosis using the TST is performed free of charge at the hospital. The interferon gamma release assay is not available at the INCan. If TST is positive, the patient is referred to the outpatient infectious diseases clinic to rule out active tuberculosis and assess the initiation of treatment for LTBI, while continuing its diagnostic and therapeutic approach by hematologists. All patients attend with a chest x-ray image that is performed as part of their hematologic approach, a positron emission tomography (PET) scan or a full-body computed tomography scan. During the Infectious Diseases consultation, patients are asked about a history of exposure to tuberculosis, consumption of non-pasteurized dairy products, smoking, alcoholism, diabetes mellitus, exposure to biomass and the presence of respiratory symptoms (cough, hemoptysis, fever and/or chills).

For the diagnosis of LTBI, the TST is considered positive if there is an induration greater than or equal to 5 mm, which is the cut-off point established for immunocompromised patients, in addition to a chest imaging without findings compatible with tuberculosis, and the absence of symptoms consistent with tuberculosis [5].

Patients who meet the above criteria are started on treatment with INH. In cases with gastric intolerance due to gastrointestinal tumors or chemotherapy, or baseline alterations in liver function tests, the initiation of treatment is postponed. This is an individualized decision made by the infectious diseases’ physicians in charge of the case.

The standard treatment used at the INCan is INH 5 mg/kg/day up to a maximum of 300 mg in tablets, plus B6 vitamin for 9 months. Patients are followed up once a month for the first month and every 2 months thereafter, with clinical and laboratory evaluations with liver function tests. In each clinical assessment, an intentional search is made for symptoms related to common adverse reactions to INH.

### Study design

We conducted a retrospective and descriptive study between 01/01/2017 and 12/31/2018 of all patients > 18 years with a hematological malignancy, and in whom a TST was performed. Sociodemographic and clinical data on the type of hematological neoplasm were collected, in addition to those related to its treatment. For the BCG vaccine record, patients were asked if they had received the vaccine, and this was confirmed by the presence of a scar on the upper arm. On the other hand, baseline values of liver function tests and those with the highest value during the period of treatment with Isoniazid were collected. The foregoing was done in order to assess tolerance and toxicity to the use of isoniazid in conjunction with chemotherapy. Hepatic toxicity was defined as elevation of transaminases five times higher than the normal upper limit, or three times higher in the presence of symptoms. In the event of liver toxicity, INH treatment was suspended. In the cases in which they presented elevation of transaminases but not in critical values, a closer follow-up was performed.

A follow-up was performed for at least 1 year after completing prophylactic treatment for LTBI, or, for patients with negative TST, 1 year after completing their oncological treatment.

### Data analysis

The prevalence of LTBI was estimated with the number of patients with LTBI diagnoses divided by the total number of patients included for analysis. The cumulative incidence of active tuberculosis was reported as the number of new cases of active tuberculosis detected during the study period divided by the total number of patients included for analysis.

A descriptive analysis was conducted. Categorical variables are expressed as frequencies, and for the quantitative variables, mean and standard deviation or median and percentiles are reported, according to the distribution of the variables, using the Kolmogorov-Smirnov test. A bivariate analysis was performed; for qualitative variables, the Chi-square or Fisher’s exact test was used, and for quantitative variables, the Student’s t-test or Mann-Whitney U test were used. All statistical analysis was conducted with the IBM SPSS program version 25.0 for the MacOS operating system in Spanish.

### Ethical considerations

The study was approved by the Institutional Review Committee “*Comité de Ética en Investigación”* (number Rev./0014/19) of the INCan. Due to the nature of the study, being observational and retrospective, the INCan’s “*Comité de Ética en Investigación*” waived the written informed consent.

## Results

**Fig. 1:**
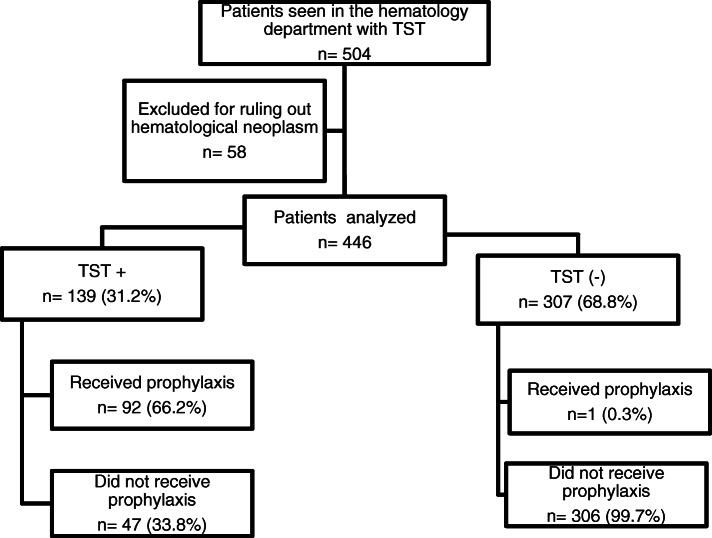
Flowchart of patients with hematological neoplasm, TST and outcomes. TST: tuberculin Skin Test

During the study period, 1372 patients were seen at the hematology department for workup, with a suspicion of hematologic neoplasms. In 96 of these patients (7%), a hematologic neoplasm was excluded. The TST was performed in 504 patients, which corresponds to 37% of all patients seen at the hematology department during the study period. Fifty-eight patients were excluded, in whom the diagnosis of hematological cancer was not confirmed. The final cohort for analysis were 446 patients (Fig. 1).

The diagnoses of the excluded patients were: non-hematological neoplasms (*n* = 31), lymphoid hyperplasia (*n* = 7), infectious pathology (*n* = 3) among which a patient with tuberculous splenitis stands out, a neoplasm was ruled out with no definitive diagnosis (*n* = 16); in one patient there were no available medical records.

Fifty-one percent of the participants were women. The median age was 54 years (40–66), 87.4% of the patients came from the central region of the country. Almost 50 % had no occupation (45.3%); 10.3% were illiterate, 13.5% did not complete elementary school, 59.9% received basic education and 17.2% had a higher degree.

Of the population studied, 40.1% had a history of smoking. The prevalence of diabetes mellitus was 15.9%, while 3.8% of patients were living with HIV. The hematologic neoplasms were: Non-Hodgkin’s lymphoma, 59.9% (*n* = 267); Hodgkin lymphoma, 17.9% (*n* = 80); multiple myeloma, 10.3% (*n* = 46); leukemia, 7.6% (*n* = 34) and other hematologic neoplasms, 4.3% (*n* = 19).

TST was positive in 139 of the 446 patients. The prevalence of latent TB was 31.2%. No active tuberculosis was documented at the time of hematological neoplasm diagnosis in any case. Table 1 shows the clinical and sociodemographic characteristics of the population studied by TST result.

**Table 1:** Clinical and sociodemographic characteristics between positive and negative TST groups

Variable	TST (+) 139 (31.2%)	TST (−) 307 (68.8%)
**Age (median, IQR)**	55 (44–67.5)	53 (38–65)
**Sex**		
Women	77 (33.9)	150 (66.1)
Men	62 (28.3)	157 (71.7)
**BCG vaccination (*n*** **= 442)**		
Yes	86 (34.4%)	164 (65.6)
No	53 (27.6)	139 (72.4)
**Educational level**		
Illiterate	16 (34.8)	30 (65.2)
Incomplete elementary school	23 (38.3)	37 (61.7)
Elementary school	24 (31.6)	52 (68.4)
Secondary school	34 (35.1)	63 (64.9)
High school or technician	23 (25.6)	67 (74.4)
Bachelor’s degree	17 (26.6)	47 (73.4)
Postgraduate studies	2 (15.4)	11 (84.6)
**Type of neoplasm**		
Hodgkin Lymphoma	11 (13.7)	69 (86.3)
Non-Hodgkin’s Lymphoma	100 (37.5)	167 (62.5)
Leukemias	12 (35.3)	22 (64.7)
Multiple Myeloma	13 (28.3)	33 (71.7)
Other haematological neoplasms	3 (15.8)	16 (84.2)
**Residence**		
Northern Region	3 (100)	N/A
Central Region	117 (30)	273 (70)
South-East Region	22 (41.5)	31 (58.5)
**Occupation**		
Household activities	58 (28.7)	144 (71.3)
Agriculture	27 (38.6)	43 (61.4)
Trades (Unqualified Labor)	37 (33.9)	72 (66.1)
Non-healthcare professional	11 (35.5)	20 (64.5)
Healthcare professional	3 (30)	7 (70)
Student	2 (15.4)	11 (84.6)
Others	10 (90.9)	1 (9.1)
**Comorbidities and exposure**		
Diabetes Mellitus	34 (47.9)	37 (52.1)
Patients living with HIV	1 (5.9)	16 (94.1)
Consumption of unpasteurized dairy	36 (72)	14 (28)
Exposure to tuberculosis cases	17 (73.9)	6 (26.1)
Alcoholism	55 (30.6)	125 (69.4)
Smoking	61 (34.1)	118 (65.9)

*BCG* Bacillus Calmette Guerin, *TST* Tuberculin Skin Test, *IQR* Interquartile range

Within the exposure history, 72% (*n* = 36) of the patients who reported a history of consumption of unpasteurized dairy products had a positive TST (OR 4.863, CI 95% 2.38–9.24, *p* < 0.0001). As expected, 74% (*n* = 17) of patients with a known exposure to TB patients had a positive TST (OR 5.074, CI 95% 1.928–13.349, *p* < 0.001).

On the other hand, only 6% (*n* = 1) of the 17 HIV positive patients of this cohort had a positive TST (OR 0.132, IC 95% 0.017–1.004, *p* = 0.029). Regarding the characteristics of the HIV patients, their median CD4 count was 164 cel/mm^3^; lymphoma diagnosis was an AIDS defining event for 41% of them, who were recently diagnosed and not on antiretroviral therapy at the moment of the TST.

Ninety-three patients received treatment for latent tuberculosis. Of these, 92 had a positive TST and it was decided to treat one patient with negative TST due to a history of direct exposure to a case of Tuberculosis. All patients with LTBI were treated with 300 mg of isoniazid daily, in addition to vitamin B6 for 9 months. Fourteen patients (15.1%) had an adverse reaction to INH: 7 had neuropathy, 4 had an increase in transaminases, and 3 had GI intolerance. A history of alcohol consumption was reported in three cases of neuropathy, one case of gastric intolerance and one case of transaminase increase. Two patients with neuropathy were smokers as well as one patient with gastric intolerance and one patient with transaminase increase. There were no associations between the exposure factors and the presence of adverse reactions.

Sixty-seven patients completed the treatment (72%). In 10 patients, there was no information available on whether or not INH was completed. Sixteen patients (17.2%) suspended treatment: 9 due to voluntary abandonment, 4 due to toxicity and medical indication (hypertransaminasemia (*n* = 3) and gastric intolerance (*n* = 1), one due to shortage of isoniazid. Active tuberculosis was documented in two patients; therefore, the regimen was changed to an antituberculous treatment with isoniazid/rifampicin/ethambutol/pyrazinamide.

The mean follow up time of the cohort was 1.77 years; 72% (*n* = 322) completed at least 1 year of follow up and 58% (*n* = 259) had at least 2 years (58%). The main reasons for not having follow up visits at one and 2 years were death (41 and 34% respectively) and lost to follow up (51 and 47% respectively).

Two cases of active tuberculosis were detected. The incidence of tuberculosis was 0.45%, corresponding to a population rate of 448 per 100,000. Both patients had LTBI and were receiving treatment with INH. The first case was a patient who had been on INH for 6 months with adequate adherence to it. While on INH, the patient presented with pneumonic symptoms, she underwent a bronchoalveolar lavage with a culture report of *Aspergillus spp* and a positive Ziehl Neelsen (ZN) stain. The culture for mycobacteria was negative. This 62-year-old patient had a diagnosis of Hodgkin’s lymphoma and had received chemotherapy and radiotherapy. She had completed 5 cycles of ABVD (adriamycin, bleomycin, vinblastine and dacarbazine) 2 months earlier and was on radiotherapy of the neck. Tuberculosis treatment was decided based on the ZN stain, the chest tomography scan and the clinical course. INH was stopped and antituberculosis treatment was started based on international guidelines with 4 drugs for 2 months (isoniazid/rifampicin/pyrazinamide/ethambutol) and two drugs for 4 more months (rifampicin/isoniazid), in addition to antifungal treatment, with a good response.

The second case of active tuberculosis was a 65-year-old male patient with a diagnosis of Non-Hodgkin’s Lymphoma on treatment, who had received 2 cycles of rituximab + CHOP (cyclophosphamide, doxorubicin, vincristine and prednisone) and was on INH prophylaxis. One month after starting INH, neck lymph node tuberculosis was diagnosed. The lymph node was cultured and reported *Mycobacterium tuberculosis.* Drug resistance testing reported susceptibility to all first line antituberculosis drugs. He received anti-tuberculous treatment for 6 months with adequate clinical response.

## Discussion

This study reports a prevalence of latent tuberculosis in patients with hematological neoplasms of 31.2%. In Mexico, the prevalence reports vary widely according to the geographic area and population evaluated. A study of migrants at the border with the United States reported a prevalence of up to 55% using a combined strategy of TST + IGRA [19]. Another study carried out at a social security institution in Mexico City with diabetic patients, found a prevalence of 51% using only TST with an induration greater than 5 mm [20]. Another study reported a prevalence of 33.5% in patients with rheumatoid arthritis using TST booster [21]. *C. Bourlon* et al., found a prevalence of 20% in a population of hematology transplant recipients using TST booster. In our case, as the patients were evaluated before starting chemotherapy, the booster was not used. In their study, *C. Bourlon* et al. report a prevalence in healthy bone marrow donors of 40% which, together with the social security institution results, probably better reflects the prevalence in the general population of Mexico [14].

The TST has been the most widely used screening method for the diagnosis of LTBI. However, given its low specificity and lower sensitivity, in patients with a high risk of reactivation of tuberculosis, such as immunosuppressed patients, a combined IGRA and TST screening has been suggested. The above, in order to increase the diagnostic efficacy and in case of positivity of any of the tests, provide treatment to these patients [10, 12, 17]. In our study, within the group of patients with negative TST, no cases of active tuberculosis were documented. Although it was not designed to evaluate the diagnostic performance of the TST, as there were no cases of reactivation in the negative TST group, it could be considered that no further diagnostic tests are necessary to improve the sensitivity of the diagnosis.

Regarding immunosuppressed patients and those undergoing cytotoxic chemotherapy, it is important to reduce the probability of drug interactions and/or suspension due to intolerance to treatment. Currently, there are shortened regimens that are recommended as first-line treatment, such as INH/rifampicin dual therapy for 3 months [1, 9] for which a similar tolerance and efficacy was reported compared to the standard regimen [1]. It has been observed that with these regimens, there is greater adherence to treatment and a similar tolerance compared to monotherapy regimens [22–25]. However, these regimens have not been studied in chemotherapy patients with the risk of interactions associated with rifampicin. In our study, 9 out of 93 patients (9.6%) abandoned treatment and this might probably be decreased with shorter schemes. More information is needed in patients with hematological malignancies in order to establish the safety and efficacy of shortened regimens and the risk of interactions with the use of rifampicin.

Most of the patients had adequate tolerance and adequate therapeutic adherence. Adverse effects occurred in 15.1%, but only 4.3% required the suspension of treatment. Neither one was a significant drug interaction demonstrated with the simultaneous use of different chemotherapy regimens during the follow-up period. Therefore, it could be considered, for now, as an adequate scheme for the treatment of LTBI in hematology-oncology patients in our institution and other similar scenarios.

There were only two cases of reactivation of tuberculosis. One patient was diagnosed a month after starting treatment for latent tuberculosis, so it is highly likely that he already had active tuberculosis and it was not diagnosed from the beginning. One of the difficulties in diagnosing patients with lymphoma is that fever, weight loss, diaphoresis, and lymphadenopathies are part of the usual hematological clinical presentation and cannot be used as a guide to diagnose tuberculosis. The other case reactivated 6 months after starting isoniazid, accompanied by a fungal co-infection by *Aspergillus sp*. Although the mycobacterial culture was not positive, the decision was to treat as tuberculosis according to clinical and radiological factors and the positive ZN stain. The fungal coinfection probably had a modulating role in the immune response of the patient and in favoring the replication and reactivation of tuberculosis.

The incidence of TB reactivation in our cohort was 0.45% in our cohort, with a population rate of 448 per 100,000, a very high rate and much higher than Mexico’s reported rate of 23 per 100,000 inhabitants in 2019 [4]. These results highlight the relevance of continuing screening and treating LTBI in high-risk populations like patients with hematological malignancies.

Another interesting fact that showed up in our study is INH and TST shortage, that can threaten TB prevention programmes as ours. This has been reported before in many countries and is a major obstacle for successful prophylactic programmes [26–28]. Our data underscore the importance of screening hematologic patients in the context of a high prevalence of LTBI and high incidence of active TB in this population, and the difficulty to achieve it.

We also found an interesting association between LTBI and consumption of unpasteurized dairy products. This finding, related to infections caused by *Mycobacterium bovis*, is frequent in developing countries [29] and has been well documented in Mexico [30], where zoonotic tuberculosis caused by *M.bovis* has shown increasing trends and reported in up to 26% of isolates [31].

This study has some limitations. It is an observational and retrospective study, with only TST as the diagnostic method to assess LTBI; no other comparisons were available because IGRAS is not available in the INCan, as in many other public institutions in Mexico. Even when patients were followed during the period of greatest risk of reactivation, it is not possible to determine whether a longer follow-up could detect more cases of tuberculosis reactivation. Another limitation is that only 37% of all hematologic patients were tested. This low TST coverage is explained by the fact that implementation of LTBI screening in the Hematology Department started slowly in 2017, and at the beginning not all patients were evaluated for TST. Secondly, during the study period, there was a shortage of TST, and no screening was done during several months. Consequently, patients in this cohort were not chosen randomly but according to the availability of the TST, and we cannot guarantee that our results reflect the whole population of our institution. We do not have the description of all the characteristics of the haematologic patients that were not tested, to compare our cohort with, but we did compare the type of diagnosis distribution of the study group with the entire hematology population for 2017 and 2018 and patients distribution is very similar.

Despite these limitations, the close and standardized follow-up of the patients, as well as the cohort studied, provides important information about LTBI in patients with hematological neoplasms in a limited resources institution.

In conclusion, the prevalence of LTBI in patients with hematologic malignancies was 31.2%, which is within the range of prevalence reported in other similar populations in Mexico but lower than other non-immunosuppressed Mexican cohorts. This is likely due to a lower sensitivity of TST in immunosuppressed patients. However, no tuberculosis reactivations were observed in the negative TST group, suggesting that TST is a good screening tool. Isoniazid as monotherapy for 9 months was generally well tolerated with a relatively low proportion of significant adverse effects. It is important to evaluate the feasibility of shortened treatments in this type of patients to improve adherence to treatment.

## Data Availability

The datasets used and/or analysed during the current study are available from the corresponding author on reasonable request.

## Abbreviations

LTBI: Latent Tuberculosis
TST: Tuberculin Skin Test
IGRAS: Interferon γ-release assays
INH: Isoniazid
PET: Positron Emission tomography
ZN: Ziehl Neelsen
